# Change of the relative line strengths due to the resonance induced population transfer between Fe XVII and FeXVI ions

**DOI:** 10.1038/s41598-019-43916-x

**Published:** 2019-05-16

**Authors:** Chensheng Wu, Xiang Gao

**Affiliations:** 1 Beijing Computational Science Research Center, Beijing, 100193 China; 2 Department of Engineering Physics, Tsinghua University, Beijing, 100084 China; 3 Center for Computational Sciences, University of Tsukuba, 1-1-1 Tennodai, Tsukuba, Ibaraki 305-8577 Japan

**Keywords:** Atomic and molecular interactions with photons, Electronic structure of atoms and molecules

## Abstract

We present a detailed study to resolve the discrepancy between the existing theoretically estimated oscillator strengths and the recently observed result from the X-ray free electron laser (XFEL) experiment performed at the Linac Coherent Light Source (LCLS) for the intensity ratio between two of the strongest emission lines from Ne-like Fe XVII (Fe^16+^) ion. By including the dynamic resonance induced population transfer due to autoionization between the coexisting Fe XVII and Fe XVI (Fe^15+^) ions in the XFEL experiment, we are able to successfully resolve this difference in theory and experiment. Further experimental works are suggested for a more detailed understanding of the dynamic resonance processes for ions.

## Introduction

The x-ray emission lines of the Ne-like Fe^16+^ ion have been observed in a variety of astrophysical objects, including the Sun, stellar coronae, elliptical galaxies, and supernova remnants^1–4^. Two of the most intense lines of the Ne-like Fe^16+^ ion are the 2p^5^3d ^1^P_1_ → 2p^6 1^S_0_ dipole emission line at 15.01 Å (3C) and the 2p^5^3d ^3^D_1_ → 2p^6 1^S_0_ inter-combination line at 15.26 Å (3D). The wavelength separation of these two lines is large enough to be resolved by spectrometers with moderate resolving power, but, is small enough so that errors in spectrometer response are relatively small^1–4^. However, the diagnostic utility of these two lines has been limited by the fact that although extensive studies have been carried out, discrepancies between the theoretical estimates and the measurements from astrophysical and laboratory sources persist^5–7^.

In a recent benchmark experiment performed at the Linac Coherent Light Source (LCLS)^5^, the highly charged Fe ions were first generated in an electron beam ion trap (EBIT) and then photo-excited by the X-ray free electron laser (XFEL). This experiment was designed to allow a direct comparison of experimental and theoretical results, excluding the effects of electron collisions. The measured weighted 3C/3D ratio was 2.61 ± 0.23, which is significantly lower than the most elaborated theoretical values at 3.4 or higher^5–10^. It was concluded that the discrepancies are due to the inaccurate atomic wave functions^5,11^. This conclusion appears to be supported by a recent configuration interaction calculation with some fine-tuning parameters^12^. However, it was argued later that the calculated ratio in ref.^12^ is unreliable due to a limited and unbalanced treatment of electron correlations^13^. Alternatively, it was proposed that other physical processes beyond those included in the atomic structure calculation of an isolated atomic system, such as the one due to the ultra-high intensities of XFEL with ultra-short pulse period, may be responsible for this discrepancy^10,14,15^. By scrutinizing the experimental conditions at the LCLS^5,11,16–18^, the intensities of the XFEL pulses are not sufficiently high to support this proposed interpretation^10,14,15^ and it is not likely that the discrepancy between theory and experiment could be attributed to the high order nonlinear effect. Thus, it is worth exploring further other possibilities to explain the experiment^5^.

Our investigation starts with a critical assessment of the theoretical intensity ratio based on an atomic structure calculation of isolated ion with a revised multi-configuration Dirac-Fock (MCDF) approach, where the quasi-complete basis scheme is adopted to optimize the atomic orbitals (AOs) using the GRASP-JT version^19,20^ based on the earlier GRASP2K codes^21,22^. In this way, the convergence of the atomic structures can be examined step by step with limited computational efforts. This approach was developed recently to study the forbidden transitions in O^+^ ion with its result within the overlap range of two available astrophysical observations and with a very small theoretical uncertainty^19^. With a converged 3C/3D ratio of 3.567 ± 0.003 from our detailed calculation, there is definitely the need to explore other possible physical effects which might bring the theoretical ratio close to the one observed recently by the XFEL experiment.

**Figure 1:**
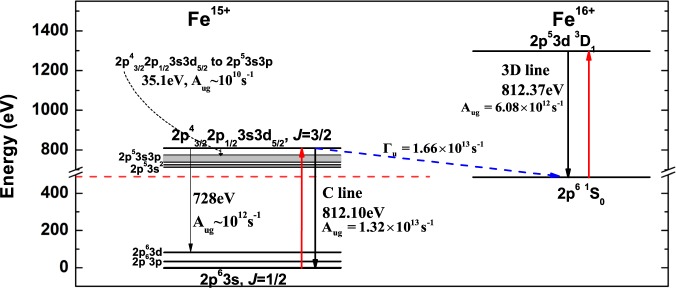
The energy levels and transitions of Fe XVI (Fe^15+^) and Fe XVII (Fe^16+^). The bold red arrows represent the excitation process of the XFEL. The bold black arrows represent the C and 3D emission lines. The thin black arrows represent some minor decay channels of the 2p^4^_3/2_2p_1/2_3s3d_5/2_ J = 3/2 resonant state. The blue dash arrow represents the autoionization from the Fe XVI. The red horizontal dash line represents the first ionization threshold at 489.31 eV of Fe XVI.

There are three known emission lines from the upper states of Fe XVI ions (Fe^15+^) to its ground state 2p^6^3s_1/2_ *J* = 1/2, i.e., line A from 2p^4^_3/2_2p_1/2_3s3d_3/2_ *J* = 3/2 state, line B from 2p^4^_3/2_2p_1/2_3s3d_3/2_ *J* = 1/2 state and line C from 2p^4^_3/2_2p_1/2_3s3d_5/2_ *J* = 3/2 state^23,24^. It is also known from the previous EBIT experiment that the C line blends with the 3D line of Fe XVII (Fe^16+^) since both have an energy close to 812 eV (indicated by the bold black arrows shown in Fig. 1)^23,24^. In the LCLS experiment, the contribution of the Fe XVI C line was subtracted from the Fe XVII spectra for the 3D line with the assumption that the emissions from Fe XVII and Fe XVI are independent^5^. It turns out that in addition to the radiative decays, this upper state of the C line from Fe XVI could also autoionize into the ground state of Fe XVII shown by the blue dash arrow in Fig. 1. Since there was a finite pulse duration of the LCLS experiment, the resulting Fe XVII in its ground state could then be excited to increase the population of the upper level of the Fe XVII 3D line and thus increases the intensity of the 3D line. On the other hand, with the XFEL photon energy at the position of the Fe XVII 3C line, there is no resonant autoionization states of the Fe XVI ion. Considering the direct photon ionization probability is several orders of magnitude lower than the resonant autoionization process, the population of the upper level of the Fe XVII 3C line will not be influenced by the Fe XVI ion and thus the intensity of 3C line will not be changed. Then a decrease of the measured 3C/3D line ratio could be expected.

In this paper, we study such resonance induced population transfer process between Fe XVI and FeXVII ions in detail, which may offer a reasonable explanation for the discrepancy between theory and experiment. We will first discuss the assessment of the theoretical 3C and 3D oscillator strengths of Fe XVII ion, and then presents the results of our study of the resonance induced population transfer process. Finally, the implications of the present work are summarized.

## Results and Discussions

### The assessment of the theoretical 3C and 3D oscillator strengths of Fe XVII ion

The calculation of the atomic structure of the Fe XVII (Fe^16+^) ion is based on the well-established full relativistic multi-configuration Dirac-Fock (MCDF) approach^21,22^. In the present study, the oscillator strengths of the transitions between the ground state and the first five J^π^ = 1^−^ excited levels of the Fe XVII are calculated together to guarantee the final convergence of the 3C and 3D lines. The quasi-complete basis scheme is adopted to optimize the atomic orbitals (AOs) using the GRASP-JT version^19,20^ based on the earlier GRASP2K codes^21,22^. In this way, the convergence of the atomic structures can be examined step by step with limited computational efforts.

We started with the atomic orbitals (AOs) with principal quantum number n = 1, 2, 3 and (n-*l*-1) nodes that are optimized by multi-configuration self-consistent-field (MCSCF) iterations to minimize the lowest 37 energy levels of 2p^6^, 2p^5^3s, 2p^5^3p, and 2p^5^3d states to form the *zeroth* level basis. With the AOs fixed up to n = 3, the pseudo AOs with n = 4 are obtained by further MCSCF iterations to optimize the statistic weights of the ground state and the first five J^P^ = 1^−^ excited levels of the Fe XVII to generate the *first* level basis. The additional electronic configurations from the 2p^6^, 2p^5^3s, 2p^5^3p, and 2p^5^3d reference configurations with n = 3 and 4 AOs (i.e., two electrons excited from the core shell and one electron excited from the valence shell) are added in our calculation to include single, double, and some important triple excitations, even those from the 2 s inner shells. In succession, by adding more and more AOs in what we termed as the quasi-complete basis, we have included in the present calculation to *sixth* level basis with n_max_ = 9.

**Figure 2:**
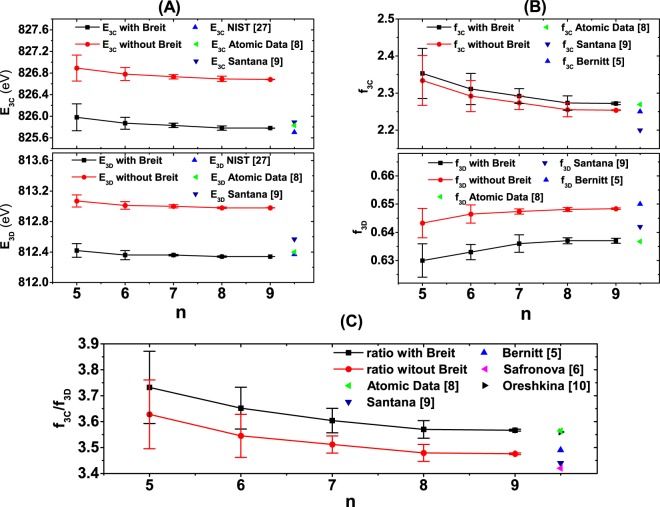
The variation of the excitation energies *E*_3C_, *E*_3D_, the oscillator strengths *f*_3C_, *f*_3D_, and the intensity ratio *f*_3C_/*f*_3D_, with and without the Breit interaction, as the size of the quasi-complete basis increases with *n* in our MCDF calculation. Comparisons with other available atomic structure calculations are also presented.

Based on these AO basis, the energy levels and corresponding transition rates are calculated by the configuration interaction (CI) method with more configurations than previous MCSCF calculations where the double excitations of the 2 s inner shell are allowed. So the CI calculations take account of all the valence- and prominent core excitation correlations, which are important for convergence. In fact, with n = 9, there are a total of over 3.7 million electronic configurations in our CI calculations. The QED corrections, especially the Breit interaction^25,26^, are added to the atomic Hamiltonian as a perturbation in the CI calculations. The Breit interaction is the most important high-order correction not only for the energy levels but also for the transition rates. The uncertainties of our calculated transition energies are estimated from the difference between CI calculations with AOs of adjacent n. The uncertainties of our calculated oscillator strengths are estimated by combining both the difference between CI calculations with AOs of adjacent n, as well as the difference between length and velocity gauge. Figure 2 shows the convergent behaviors of excitation energies *E*, oscillator strengths *f* and the oscillator strengths ratio of 3C and 3D with estimated uncertainties of various AO bases, compared with some reference data^5,6,8–10,27^. Table 1 presents the final calculation values using the quasi-complete basis (i.e., AOs with n = 9), compared with some reference data^8,27^.

**Table 1:** The calculated excitation energies (in eV) of the first five J^P^ = 1^−^ levels from the ground state of Fe XVII and their oscillator strength.

Configuration	E^DC a^	E^DC+Breit b^	E^NIST c^	f^DC a^	f^DC+Breit b^	f^Ref d^
(2p^5^)_3/2_3s_1/2_	727.444(5)	727.018(5)	727.139	0.123(1)	0.123(1)	0.122
(2p^5^)_1/2_3s_1/2_	739.681(5)	738.941(6)	739.054	0.101(1)	0.103(1)	0.101
(2p^5^)_3/2_3d_3/2_	802.835(3)	802.271(3)	802.401	0.010(1)	0.010(1)	0.010
(2p^5^)_3/2_3d_5/2_ (3D)	812.982(1)	812.343(1)	812.369	0.648(1)	0.637(1)	0.637
(2p^5^)_1/2_3d_3/2_ (3C)	826.685(2)	825.782(2)	825.700	2.254(1)	2.272(4)	2.269

^a^The calculations with Dirac-Coulomb Hamiltonian.

^b^The calculations with Dirac-Coulomb Hamiltonian, Breit interactions and other high order QED corrections.

^c^The experimental values from NIST database^27^.

^d^The MCDF calculation results from ref.^8^.

In addition to the excellent agreement between the length and velocity results of our theoretical oscillator strength *f*_3C_ and *f*_3D_, our calculated excitation energy E, *f*_3C_, *f*_3D_, and the ratio *f*_3C_/*f*_3D_ are indeed influenced by one of the most important QED corrections, the Breit interaction^25,26^. By including the Breit correction, the excitation energies from the current calculation are less than 0.02% from the NIST data^27^. The *f*_3C_/*f*_3D_ ratio changes from a value of 3.476 ± 0.003 to 3.567 ± 0.003 since the value of *f*_3C_ increases while the value of *f*_3D_ decreases after the Breit interaction was taken into account. With a converged ratio from our detailed calculation in close agreement with other existing atomic structure calculations^5–10^, we then conclude that other physics must be responsible for the discrepancy between previous theory and experiment.

### Theoretical model for resonance induced population transfer process

To study the resonance induced population transfer process between the Fe XVI and Fe XVII ions, we note first that, quantitatively, the time scale of the emission process of photon is usually of the order of 100–1000 fs, whereas the electron processes, such as the collision ionization (CI), the radiative recombination (RR), the dielectronic recombination (DR), and the excitation autoionization (EA) in the EBIT are typically of the order of 10^−3^ s^28–30^. Second, the XFEL photon energy was continuously scanned with a repetition rate of 120 Hz (Supplementary of ref.^5^), which means after the interaction with the XFEL, the ions in the EBIT have adequate time to return to their equilibrium stable state. Therefore, we could decouple those electron processes from the photon process when we discuss this XFEL photon related resonance induced population transfer process. By including the dominant decay channel (i.e., to the ground state of Fe XVI, which will be discussed later) and the autoionization of Fe XVI into the ground state of Fe XVII, the rate equations for the population densities for the ground and excited states of Fe XVI and FeXVII ions with incident XFEL photon energies $\hslash {\rm{\omega }}$ around 812 eV (the C and 3D lines) could then be expressed as:

1A $$\begin{array}{rcl}\frac{d}{dt}{N}_{g}^{F{e}^{15+}}(t) & = & {N}_{u}^{F{e}^{15+}}(t){B}_{ug}^{F{e}^{15+}}({\omega }_{C})\rho (\omega )+{N}_{u}^{F{e}^{15+}}(t){A}_{ug}^{F{e}^{15+}}({\omega }_{C})\\  &  & -{N}_{g}^{F{e}^{15+}}(t){B}_{gu}^{F{e}^{15+}}({\omega }_{C})\rho (\omega ),\,\end{array}$$

1B $$\begin{array}{rcl}\frac{d}{dt}{N}_{u}^{F{e}^{15+}}(t) & = & -{N}_{u}^{F{e}^{15+}}(t){B}_{ug}^{F{e}^{15+}}({\omega }_{C})\rho (\omega )-{N}_{u}^{F{e}^{15+}}(t){A}_{ug}^{F{e}^{15+}}({\omega }_{C})\\  &  & +{N}_{g}^{F{e}^{15+}}(t){B}_{gu}^{F{e}^{15+}}({\omega }_{C})\rho (\omega )-{N}_{u}^{F{e}^{15+}}(t){\Gamma }_{u}^{A},\end{array}\,$$

1C $$\begin{array}{rcl}\frac{d}{dt}{N}_{g}^{F{e}^{16+}}(t) & = & {N}_{u}^{F{e}^{16+}}(t){B}_{ug}^{F{e}^{16+}}({\omega }_{3D})\rho (\omega )+{N}_{u}^{F{e}^{16+}}(t){A}_{ug}^{F{e}^{16+}}({\omega }_{3D})\\  &  & -{N}_{g}^{F{e}^{16+}}(t){B}_{gu}^{F{e}^{16+}}({\omega }_{3D})\rho (\omega )+{N}_{u}^{F{e}^{15+}}(t){\Gamma }_{u}^{A},\end{array}$$

1D $$\begin{array}{rcl}\frac{d}{dt}{N}_{u}^{F{e}^{16+}}(t) & = & -{N}_{u}^{F{e}^{16+}}(t){B}_{ug}^{F{e}^{16+}}({\omega }_{3D})\rho (\omega )-{N}_{u}^{F{e}^{16+}}(t){A}_{ug}^{F{e}^{16+}}({\omega }_{3D})\\  &  & +{N}_{g}^{F{e}^{16+}}(t){B}_{gu}^{F{e}^{16+}}({\omega }_{3D})\rho (\omega ),\,\end{array}$$

where the transition rate *A*_*ug*_ is the Einstein *A* coefficients from the upper level to the lower level, *B*_*ug*_ and *B*_*gu*_ are the Einstein *B* coefficients with ${B}_{ug}=({\pi }^{2}{c}^{3}/\hslash {{\rm{\omega }}}^{3}){A}_{ug}$, and ${{\rm{\Gamma }}}_{u}^{A}\,$is the autoionization rate of Fe XVI ion. $\rho (\omega )={I}_{p}/(c\Delta \omega \sqrt{2\pi })\exp [-{(\omega -{\omega }_{k})}^{2}/2\Delta {\omega }^{2}]$, where *I*_*p*_ is the peak intensities of XFEL around energy *ω*_*k*_, Δω is the line width of the XFEL with the assumption of Gaussian profile*, ω*_*k*_ is the resonant energy of the respective transition, and *c* is the speed of light^31^. The initial conditions of the equations are ${N}_{u}^{F{e}^{16+}}(t=0)=0,\,{N}_{u}^{F{e}^{15+}}(t=0)=0$ with ${N}_{g}^{F{e}^{15+}}(t=0)/{N}_{g}^{F{e}^{16+}}(t=0)$ as the concentration ratio between Fe XVI and Fe XVII ions in the EBIT. For other XFEL photon energies between 810 eV and 830 eV where the Fe XVII and Fe XVI lines are well separated, the rate equations are simpler. More specifically, around the resonant energies of the Fe XVI A and B lines, only Eqs (1A) and (1B) are needed (with *ω*_*c*_ replaced by *ω*_*A*_ and *ω*_*B*_, respectively), whereas for the Fe XVII 3C line, only Eqs (1C) and (1D) are needed (without the last autoionization term and with *ω*_3*D*_ replaced by *ω*_3*C*_).

After solving Eq. (1) at a specific XFEL photon energy *ω*_0_ with peak intensity *I*_*p*_ for time-dependent population of the upper level of Fe XVII ${N}_{u}^{F{e}^{16+}}(t)$ and Fe XVI ${N}_{u}^{F{e}^{15+}}(t)$, the total fluorescence photon number ${N}_{ph}^{i}({I}_{p},{\omega }_{0})$ is given by,

2 $${N}_{ph}^{i}({I}_{p},{\omega }_{0})={\int }_{0}^{\tau }{N}_{u}^{i}(t){A}_{ug}^{i}({\omega }_{0})dt+{N}_{u}^{i}(\tau ){\beta }^{i},$$

where *τ* is the incident XFEL pulse duration, *i* represents the Fe XVI or Fe XVII ion respectively and *β*^*i*^ is the transition branching ratio, which equals 1 for Fe XVII and ${A}_{ug}^{F{e}^{15+}}/({A}_{ug}^{F{e}^{15+}}+{\Gamma }_{u}^{A})$ for Fe XVI. The first term represents the emitted photons during the pulse period, the second is the remaining emitted photons at the end of the pulse. Note that only the spontaneous emission is relevant for the fluorescence because the photons emitted from stimulated radiation have the same direction as incident laser, which are not detected by the detectors perpendicular to the incident laser^5^. The anisotropic angular distributions are also taken into account in present simulations.

### The parameters used in the simulation

All required transition rates between 810 eV and 830 eV for the Fe XVII ions have already been obtained from the extended MCDF calculation discussed earlier. For the Fe XVI ion, the transition rates are calculated with the well-established atomic structure theories such as the relativistic eigenchannel R-matrix method detailed elsewhere^20,32^. In particular, for the autoionization rate, our calculation has taken into account fully the interaction between various resonant states and the continua. The relevant transition and autoionization rates are, respectively, 0.87 × 10^13^ s^−1^ and 3.92 × 10^13^ s^−1^ for the A line, 2.45 × 10^13^ s^−1^ and 2.13 × 10^12^ s^−1^ for the B line, and 1.32 × 10^13^ s^−1^ and 1.662 × 10^13^ s^−1^ for the C line. In addition, the radiative decay from the upper 2p^4^_3/2_2p_1/2_3s3d_5/2_ *J* = 3/2 state is dominated by C line with its decay rate at least one order of magnitude greater than the other decays channels (including various cascade decay processes) as shown in Fig. 1. At specific photon energy, only the dominant decay channel and the autoionization of Fe XVI into the ground state of Fe XVII (if it contributes to the change in the Fe XVII population) are included when solving the rate equations.

We then examine briefly the relevant XFEL parameters in the LCLS experiment^5^ which we will apply in the numerical simulation presented later. For the line width parameter Δω of the XFEL photons, we use the value of 0.4 eV in all our simulations, which will result in a good match with the experimental spectra resolution^5^. The photon intensity *I*_*p*_ used in the simulations can be obtained from pulse energy *P*_*E*_, pulse duration *τ* and the effective focal area ${\sigma }_{d}={f}_{d}^{2}$ with *f*_*d*_ the focal diameter of the photon beam by ${I}_{p}={P}_{E}/(\tau {\sigma }_{d})$. Unfortunately, these three parameters were not well defined in the experiment^5^, we can only infer the ranges of them from the LCLS specifications^11,16–18,33,34^. The raw X-ray pulse energy before the monochromator is about 1–4 mJ^11,16^. The final pulse energies reaching the experimental end stations after the monochromator range from 0.8 μJ for 500 eV photons to 0.48 mJ for 1000 eV photons^33,34^. According to ref.^11^, the pulse duration *τ* for the XFEL ranges from 50 to 500 fs. For the focal diameter *f*_*d*_ of the photon beam, the unfocused beam size is in the range 1–3 mm^17,33,34^ with the focusing capability to about 2 μm^17,33^. Since it was stated in the Supplementary of ref.^5^ that “a very weakly focused photon beam” was used in the LCLS experiment and it is also reasonable to expect an adequate overlap between the XFEL photons with the EBIT ions in the diameter 0.5 mm (Supplementary of ref.^5^), we assume a larger *f*_*d*_ (e.g., from 25 μm to 250 μm) in our numerical simulations, which may be closer to the actual experiment conditions.

### The results of pure Fe XVI ion

**Figure 3:**
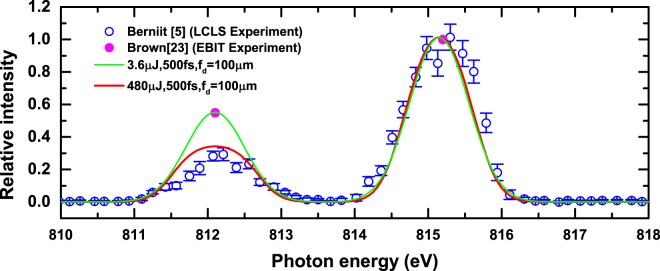
Comparison of the experimentally observed spectra of Fe XVI ions to our simulated spectra with (1) *P*_*E*_ = 3.6 μJ, *τ* = 500 fs, and *f*_*d*_ = 100 μm and (2) *P*_*E*_ = 0.48 mJ, *τ* = 500 fs, and *f*_*d*_ = 100 μm.

To examine the effect of the autoionization to the upper state population of the Fe XVI C line, we first focus our study on the simulation of the Fe XVI only spectrum shown in Fig. (3B) of ref.^5^ by solving Eqs (1A) and (1B). Figure 3 compares the experimentally observed spectra to our simulated spectra at two sets of XFEL pulse parameters. At lower pulse energy, our simulated spectrum is in good agreement with the previous EBIT experiment using electron excitation by Brown *et al*.^23^. Its higher intensity ratio of over 0.5 between the C and B lines mainly reflects the branching ratio between the C and B lines that is determined by the atomic transition rates, with little population loss of the C line. On the other hand, as expected, the intensity ratio of the C and B lines decreases from over 0.5 at lower XFEL power to 0.3 at higher XFEL power with a substantial population loss of the upper state of C line due to the autoionization and thus a much closer agreement with the observed spectrum from Fig. (3B) of ref.^5^. Accordingly, what is shown in Fig. 3 may offer the possibility of an experimental verification of the reliability of the theoretically estimated autoionization rate employed in our simulation should the values of pulse energy, duration and effective focal diameters are measured. The result in Fig. 3 also indicates that, in the XFEL pulse duration, the autoionized Fe XVII ion did not effectively survive in the EBIT under the condition to produce “pure” Fe XVI ion. On the other hand, under the EBIT condition where the Fe XVI and Fe XVII ions can co-exist, it is expected that the autoionized Fe XVII ion should survive in the EBIT during the XFEL pulse. This slight difference in the EBIT for the two measurement implies one should be careful about the subtraction of the C line contribution from the mixed spectra for the intensity of Fe XVII 3D line.

### The results of the mixed Fe XVI and Fe XVII ions

**Figure 4:**
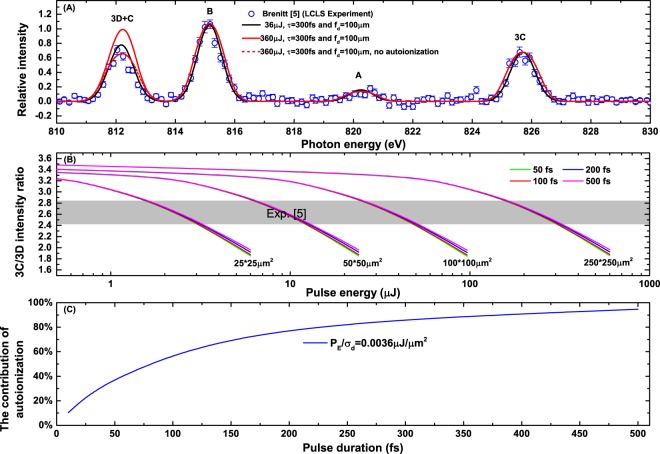
The resonance induced population transfer effects of the mixing Fe^15+^ and Fe^16+^ ions. (**A**) Comparison of the experimentally observed spectra of the mixing Fe^15+^ and Fe^16+^ ions to our simulated spectra. (**B**) The intensity ratios of 3C/3D by considering the autoionization of Fe^15+^ ions with various XFEL pulse parameters. (**C**) The contribution of autoionization for 3C/3D ratio of 2.6 with various pulse durations.

We now turn our discussion to the combined Fe XVI and Fe XVII system. To simulate the experimental spectra of the mixture (Fig. (3A) of ref.^5^), the initial relative abundance between Fe XVII and Fe XVI ions in the EBIT is required. Since the autoionization of the Fe XVI B line is almost negligible, we can use the intensity ratio of Fe XVI B line and Fe XVII 3C line to determine this relative abundance, which is about 1.5 from ref.^5^. After considering the angular distribution differences^23^ of these two lines, the ratio is corrected to 1.73. Then, by solving the related rate equations discussed above, the ion abundance of Fe XVI and Fe XVII is determined to be around 5:1. Figure 4(A) compares our simulated spectra with the experimentally observed spectrum. We start our discussion by first examining two spectra simulated at pulse parameters of *P*_*E*_ = 360 μJ, *τ* = 300 fs and *f*_*d*_ = 100 μm with and without autoionization shown by the red solid and red dash curves, respectively. As expected, these two simulated spectra are nearly identical for the B and A lines for the Fe XVI ion and the 3C line for the Fe XVII ion. In contrast, the substantial difference between these two curves for the combined 3D and C lines near 812 eV demonstrates clearly the effect of the population transfer from the upper state of the Fe XVI C line to the ground state of Fe XVII due to autoionization. Also shown in Fig. 4(A) by the dark solid curve is our simulated spectrum with two same pulse parameters *τ* = 300 fs and *f*_*d*_ = 100 μm, but, at smaller energy *P*_*E*_ = 36 μJ. The fact that this simulated spectrum is in very close agreement with the observed spectra supports strongly the reliability of the atomic data (such as the transition rates for Fe XVII and Fe XVI ions) and the simulation based on the rate equations employed in the present study. By taking into account the autoionization from the upper state of Fe XVI to the ground state of Fe XVII, as shown by the solid dark curve in Fig. 4(A), our simulated line ratio ${\rm{R}}={N}_{ph}^{F{e}^{16+}}({I}_{p},{\omega }_{3C})/{N}_{ph}^{F{e}^{16+}}({I}_{p},{\omega }_{3D})$, where ${N}_{ph}^{F{e}^{16+}}({I}_{p},{\omega }_{3C})$ and ${N}_{ph}^{F{e}^{16+}}({I}_{p},{\omega }_{3D})$ are calculated from Eq. (2) with the complete solution of Eq. (1), is expected to yield the intensity ratio of the 3C and 3D lines observed experimentally. Figure 4(B) presents the variation of the simulated 3C/3D intensity ratio with various possible XFEL parameter combinations. It is interesting to note that the 3C/3D ratio decreases as the pulse energy increases with the same focal diameter *f*_*d*_ due to the stronger autoionization effect. Similarly, the 3C/3D ratio decreases significantly, again i.e., with stronger autoionization effect, as photon intensity *I*_*p*_ increases at the same pulse energy *P*_*E*_ with smaller effective focal diameter *f*_*d*_. On the other hand, with the same pulse energy and focal diameter, the 3C/3D ratio does not change significantly with pulse duration varying from 50 fs to 500 fs, which suggests that the ratio depends mostly on the ${P}_{E}/{\sigma }_{d}$.

For the sake of further analysis and considering the lifetime of the Fe XVI C line about 60 fs which is comparable with some XFEL pulse durations it is worth to separate the contribution of resonance induced population transfer effect from our simulations by, ${P}^{{\rm{AI}}}=({R}^{{\rm{no}}-{\rm{AI}}}-{R}^{{\rm{AI}}})/({R}^{{\rm{atomic}}}-{R}^{{\rm{AI}}})$, where ${R}^{{\rm{atomic}}}=3.567$ is the calculated 3C/3D ratio of the isolated Fe XVII ion; *R*^AI^, *R*^no−AI^ are the simulated line ratio, with and without the autoionization terms in Eq. (1), respectively. Figure 4(C) shows the *P*^AI^ of a fixed ${P}_{E}/{\sigma }_{d}$ value that corresponds to the experimental measured ratio of 2.61 with various pulse durations. We can clearly see that with the pulse duration time less than 20 fs, or, substantially smaller than the lifetime of the Fe XVI C line, the contribution from the autoionization of the Fe XVI ion is small. For those XFEL parameters, the decrease of 3C/3D ratio should be attributed to the non-equilibrium effects discussed in refs^14,15^. By contrast, with the pulse durations longer than 90 fs (in consistent with the typical LCLS X-ray pulse parameters^11,16,17,33^), the resonance induced population transfer effect contributes more than a half. For example, for the pulse duration of 400 fs, where the high order nonlinear effects should be small, the contribution of the resonance induced population transfer effect is dominant, with *P*^AI^ over 90%.

### The influence of the stochastic substructure of the XFEL pulses on theoretical simulations

**Figure 5:**
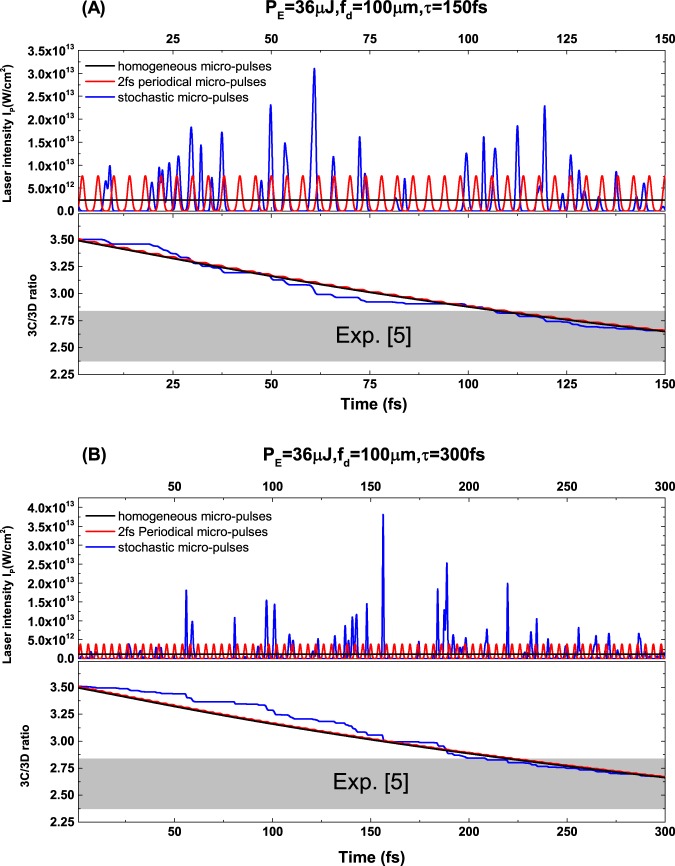
The variation of the 3C/3D ratio within the pulse duration. (**A**) The results for XFEL pulse parameter with pulse energy *P*_*E*_ = 36 µJ, focal diameter *fd* = 100 µm, and pulse duration τ = 150 fs. The upper panel exhibits the three different pulse substructures: homogeneous micro-pulses in black, the periodical micro-pulses with the period of 2 fs in red, and the stochastic micro-pulses in blue. The lower panel exhibits the simulated time dependent 3C/3D ratio with respect to these three pulse substructures. (**B**) The results for another XFEL pulse with the same *P*_*E*_ and *f*_*d*_ parameters as those in (**A**), but with a different pulse duration *τ* = 300 fs.

Due to the stochastic nature of the self-amplified stimulated emission (SASE) process, the pulse intensity of the XFEL is not homogeneous, which consists of sharp and stochastic individual pulses of 1–2 fs in duration with similar sized random gaps between the spikes^35–37^. We now discuss the influence of these stochastic substructure of the XFEL pulses on our simulation results. As an illustration example, we choose *P*_*E*_/*σ*_*d*_ = 0.0036 μJ/μm^2^ for the XFEL pulses [the same with the one in Fig. 4(C)]. With this pulse parameter, three typical substructures were considered in the simulations, i.e., (1) the homogeneous micro-pulses (constant photon intensity), (2) the periodical micro-pulses with the period of 2 fs, and (3) the stochastic micro-pulses. Figure 5(A) shows the corresponding results for the XFEL pulse with the duration τ = 150 fs. For these three different pulse substructures, the time variations of the simulated 3C/3D line ratio are almost identical, especially at the end of the pulse, which are all in good agreement with the experimental measurement^5^. Figure 5(B) shows the results for another different XFEL pulse with 300 fs duration. Similarly, the behavior of the 3C/3D ratio is also nearly independent with different pulse substructures. Therefore, we can make the conclusion that the final 3C/3D ratio mainly depends on the ratio of ${P}_{E}/{\sigma }_{d}$, the stochastic substructure of the XFEL pulses should have negligible effects on the 3C/3D ratio.

## Conclusion

In summary, with an extended large scale full relativistic configuration interaction calculation including the quantum electrodynamics (QED) term such as the Breit interaction, we first came to the conclusion that the reliability of the theoretical results for isolated Fe XVII ions from the present calculation and other earlier atomic structure calculations may not be the cause for the discrepancy between the experimentally observed intensity ratio and the theoretical estimates. Since it is known that the energies of the Fe XVII 3D line and the Fe XVI C line are both close to 812 eV and the pulse length of the XFEL experiment is sufficiently longer than the time scale of the autoionization process transferring the upper state of the Fe XVI ion to the ground state of Fe XVII ion, we decided to focus our investigation on the dynamic resonance induced population transfer from Fe XVI to Fe XVII. By solving the relevant rate equations, we are able to generate theoretically simulated spectra as well as the intensity ratio in agreement with the experimental spectra from the LCLS experiment shown in Fig. 3 of ref.^5^. Note that we have also considered the influence of the plasma environment in the EBIT. Although the plasma screening will induce a substantial decrease in the ratio 3C/3D, it could not be the one responsible for the disagreement between theory and experiment due to the low electron densities^38,39^.

In addition to resolving the discrepancy on intensity ratio discussed above, the conclusion from the present study may be examined by either performing the measurements of Fe XVI and Fe XVI/Fe XVII mixture under the well characterized XFEL intensity or with additional experiments for other Ne-like ions in the absence of the resonance induced population transfer. For example, based on our simulations, the measured ratio mainly depends on the ratio of the pulse energy and the effective focal area, i.e., *P*_*E*_/*σ*_*d*_, for which some diagnostics are being developed at the LCLS^40,41^. On the other hand, some attenuators^18^ may be used to reduce the XFEL intensity where the resonance induced population transfer effect is negligible, or alternatively, one can extract the pure Fe XVII ion from the EBIT known as the Electron Beam Ion Sources (EBIS)^42^ for the experiment.

## Data Availability

The datasets generated during and/or analysed during the current study are available from the corresponding author on reasonable request.
